# Biomarkers of blood cadmium and incidence of cardiovascular events in non-smokers: results from a population-based proteomics study

**DOI:** 10.1186/s12014-019-9231-7

**Published:** 2019-05-15

**Authors:** Yan Borné, Björn Fagerberg, Gerd Sallsten, Bo Hedblad, Margaretha Persson, Olle Melander, Jan Nilsson, Marju Orho-Melander, Lars Barregard, Gunnar Engström

**Affiliations:** 1 Department of Clinical Sciences in Malmö, CRC, Lund University, Jan Waldenströms gata 35, 205 02 Malmö, Sweden; 2 Department of Molecular and Clinical Medicine, Wallenberg Laboratory for Cardiovascular and Metabolic Research, Sahlgrenska University Hospital, University of Gothenburg, 413 45 Gothenburg, Sweden; 3 Department of Occupational and Environmental Medicine, Sahlgrenska University Hospital, University of Gothenburg, 413 45 Gothenburg, Sweden

**Keywords:** Proteomics study, Cadmium, Non-smokers, CVD, Cohort study

## Abstract

**Background:**

Cadmium is a toxic metal with multiple adverse health effects, including risk of cardiovascular disease (CVD). The mechanistic link between cadmium and CVD is unclear. Our aim was to examine the associations between blood cadmium (B-Cd) and 88 potential protein biomarkers of CVD.

**Methods:**

B-Cd and 88 plasma proteins were measured in a community-based prospective cohort, the Malmö Diet and Cancer study. The primary analysis was performed in never smokers (n = 1725). Multiple linear regression was used with adjustments for age and sex, and correction for multiple comparisons using the false discovery rate method. Proteins significantly associated with B-Cd were replicated in long-term former smokers (n = 782). Significant proteins were then studied in relation to incidence of CVD (i.e., coronary events or ischemic stroke) in never smokers.

**Results:**

Fifteen proteins were associated with B-Cd in never smokers. Eight of them were replicated in long-term former smokers. Kidney injury molecule-1, fibroblast growth factor-23 (FGF23), tumor necrosis factor receptor-2, matrix metalloproteinase-12, cathepsin L1, urokinase plasminogen activator receptor, C-C motif chemokine-3 (CCL3), and chemokine (C-X3-C motif) ligand-1 were associated with B-Cd both in never smokers and long-term former smokers. Except for CCL3 and FGF23, these proteins were also significantly associated with incidence of CVD.

**Conclusions:**

B-Cd in non-smokers was associated with eight potential plasma biomarkers of CVD and kidney injury. The results suggest pathways for the associations between B-Cd and CVD and kidney injury.

## Background

Cadmium is a toxic non-essential metal with multiple adverse health effects [1]. The main sources of cadmium are tobacco smoking and diets containing grains and vegetables from contaminated soils [2]. Cadmium concentrations are often several-fold higher in smokers compared to non-smokers. In humans, cadmium accumulates mainly in the kidneys (about 50%), liver (15%) and muscle (20%) [1, 2]. High concentrations are also found in erythrocytes and concentrations in plasma are very low. There is no efficient excretion mechanism of cadmium; only small amounts are excreted in urine. Elimination is therefore very slow with a half-life of 10–30 years [2]. Blood cadmium (B-Cd) is considered as a valid measure of body burden of cadmium during steady state.

It is well established that exposure to high concentrations of cadmium can cause kidney injury [1, 3]. Several studies suggest that cadmium also could cause atherosclerosis and cardiovascular diseases (CVD). Studies from the Strong Heart Study and the National Health and Nutrition Examination Study (NHANES) have shown associations between blood or urine cadmium and CVD or cardiovascular death [4–6]. We have recently shown similar relationships for B-Cd in cross-sectional and prospective studies of the Malmö Diet and Cancer-cardiovascular cohort (MDC-CC) in Sweden [7–9]. In these studies, B-Cd has been associated with prevalence of carotid plaque and increased incidence of coronary events and stroke. Both in US and Sweden, the increased cardiovascular risk has been observed for individuals in the top 20–25% of the distribution of B-Cd concentrations, i.e., above 0.5 µg/L [4, 7, 10].

The causal link between B-Cd and CVD is unclear at present. However, it has been proposed that cadmium has proinflammatory effects [11, 12] and that cadmium could inhibit proliferation of vascular smooth muscle cells [13]. Experimental studies have reported increased apoptosis and increased expression of proteolytic enzymes in endothelial cells exposed to cadmium [14, 15].

In order to search for possible mechanisms linking cadmium exposure to CVD, our aim was to examine the relationship between B-Cd and a panel of circulating proteins known or suggested to be related to CVD pathology. Smoking is a major source of cadmium and B-Cd concentrations are often several-fold increased in smokers [2]. It is also well known that smoking has very strong effects on the plasma protein concentrations [16]. We therefore excluded smokers to eliminate the confounding effects of smoking from the analysis. The relationship between B-Cd and protein biomarkers was explored in life-long never smokers from the MDC-CC, and significant findings were replicated in former smokers who had been smoke-free for 15 years or more. For plasma proteins significantly associated with B-Cd both in never smokers and long-term former smokers, we also explored their associations with incidence of CVD.

## Methods

### The MDC cohort

During 1991 and 1996, all men and women in the city of Malmö, Sweden, born between 1923 and 1950, were invited to participate in the MDC (participation rate was 41%), which included a health examination at a screening center [17]. During 1991–1994, a random 50% of the participants in the MDC were included in a cardiovascular sub-study (MDC-CC) (n = 6103) [7, 18]. Blood samples were taken and erythrocytes were stored in − 80 °C until analysis [19]. Smoking habits were assessed in a self-administrated questionnaire. Fiber intake was assessed using a 168-item food frequency questionnaire, a 7-day food diary and a 1-h diet interview.

Cadmium was analyzed in erythrocytes using inductively coupled plasma mass spectrometry operating in the helium collision cell mode [7, 9]. The imprecision was 9.6%, calculated as the coefficient of variation for 50 duplicate samples (mean 0.43 µg/L). The detection limit was 0.02 µg/L. B-Cd was calculated by multiplying erythrocyte cadmium with hematocrit.

Information about plasma proteins was available in 4742 participants and of those, 4232 had data on plasma proteins and B-Cd. Out of these 1725 were never smokers and 1414 were long-term former smokers. Never smokers were used for primary analysis. Of the long-term former smokers, 782 had been smoke-free for 15 years or more and this group was used to replicate significant findings from the analysis of never smokers. The rationale for selecting this group was that a long abstinence is needed for cadmium levels and inflammatory markers to be reduced after smoking cessation [20]. By using this approach, we minimized the proinflammatory effect remaining after smoking cessation.

Ninety-two human protein biomarkers have been measured using Proseek Multiplex CVD I 96 × 96 Kit (Olink Bioscience, Uppsala, Sweden) based on the Proximity Extension Assay technology with the Fluidigm BioMark HD real-time PCR platform in 54 chip runs [21]. The output unit is presented as normalized protein expression (NPX) arbitrary units (AU) on a log2 scale. Limit of detection (LOD) is defined as 3× standard deviations (SD) above background based on the negative controls in each run. Intra- and inter-assay coefficients of variation for the various proteins are presented on www.olink.se [21].

**Fig. 1:**
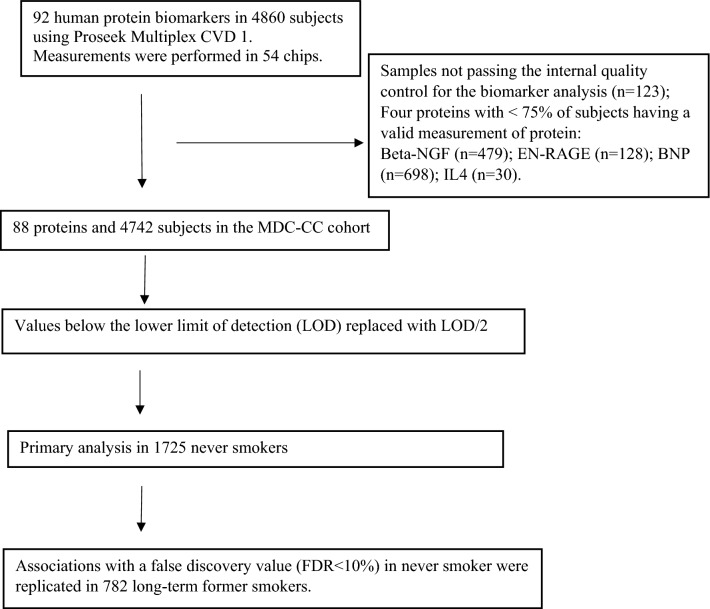
Flow chart

We excluded samples not passing the internal quality control for the biomarker analysis (n = 123). We also excluded four proteins, for which less than 75% of subjects had a valid measurement: beta-nerve growth factor (Beta-NGF); Extracellular Newly Identified RAGE-Binding (EN-RAGE); B-type natriuretic peptide (BNP); Interleukin-4 (IL4). Hence, there was information of 88 proteins (Fig. 1). The individuals with protein values below the LOD were replaced with LOD/2.

Incidence of CVD includes coronary event and ischemic stroke. An ischemic stroke diagnosis was defined as codes 434 or I63, according to the International Classification of Diseases (ICD), 9th or 10th revision, respectively. A coronary event was defined as a fatal or non-fatal myocardial infarction (ICD-9 and ICD-10 codes 410 and I21, respectively) or death due to ischemic heart disease (codes 411, 412 and 414 (ICD-9) or I22-I25 (ICD-10). Never smokers without history of stroke or coronary events (n = 1705) were followed from the baseline examination until incident first-onset of ischemic stroke or coronary event, emigration from Sweden, death or December 31st, 2014, whichever came first. The Swedish Hospital Discharge Register (SHDR) and the Swedish Cause of Death Register were used.

### Statistics

Multiple linear regressions were performed (one protein at a time), with B-Cd, age and sex as independent variables, and protein level as dependent variable. All proteins were standardized before analysis (mean = 0, SD = 1), to facilitate comparisons between proteins. The results are presented as beta coefficients (95% confidence interval (CI)) and represent the increase in SD of the log transformed protein level per 1 µg/L increment of B-Cd. The primary analysis was performed in never smokers (n = 1725). Associations passing the correction for multiple comparisons (false discovery rate, FDR q < 10%) in never smokers were replicated in long-term former smokers (n = 782). For proteins that were replicated in long-term former smokers, we also adjusted for intake of fiber in a sensitivity analysis, since fiber is an important source of dietary cadmium. A p value < 0.05 was considered as statistically significant. Cox’ proportional hazard regression was used to assess the association of baseline proteins with incident CVD. Hazard ratios (HR) and corresponding 95% CIs were calculated per 1 SD increment of protein biomarkers.

IBM SPSS version 22 (IBM Corp.) or Stata software version 12.0 (StataCorp) was used for analyses.

## Results

The characteristics of the study samples are presented in Table 1. The range of B-Cd in never smokers was 0.02–2.31 µg/L. B-Cd (µg/L) (median 0.20, mean 0.23, SD 0.16) was similar in never smokers and long-term former smokers.

**Table 1:** The characteristics of the study samples and excluded individuals

	Never smokers (n = 1725)	Long-term former smokers (n = 782)	Excluded current smokers (n = 1088)
B-Cd (µg/L) mean (SD)/median	0.23 (0.16)/0.20	0.23 (0.16)/0.20	1.04 (0.71)/0.90
Age (years)	58.0 (5.8)	58.1 (5.9)	56.2 (5.8)
Sex (male, n, %)	497 (28.8)	399 (51.1)	440 (40.4)
Diabetes mellitus [n (%)]	113 (6.6)	67 (8.6)	74 (6.8)
BMI (kg/m^2^)	25.7 (3.9)	26.0 (3.8)	24.8 (3.9)
LDL (mmol/L)	4.21 (0.98)	4.12 (0.95)	4.18 (0.99)
HDL (mmol/L)	1.43 (0.37)	1.39 (0.37)	1.36 (0.37)
hsCRP (mg/L)^a^	1.20 (0.6–2.4)	1.20 (0.6–2.3)	1.60 (0.8–3.4)
Anemic status (n, %)	45 (2.6)	20 (2.6)	20 (1.8)
Systolic blood pressure (mmHg)	141 (18.5)	141 (17.7)	138 (19.1)
Low education [n (%)]	765 (44.3)	374 (42.9)	517 (47.6)
Dietary intake of fiber (g/day)^a^	20.4 (16.6–25.0)	21.4 (17.0–26.3)	18.6 (14.7–23.6)

Values expressed are means (±SD) or percentages unless specified elsewise

*B*-*Cd* blood cadmium, *BMI* body mass index, *LDL* low-density lipoprotein, *HDL* high density lipoprotein, *hsCRP* high-sensitive C-reactive protein

^a^Median (25–75%)

### Cadmium and protein biomarkers in never smokers

**Fig. 2:**
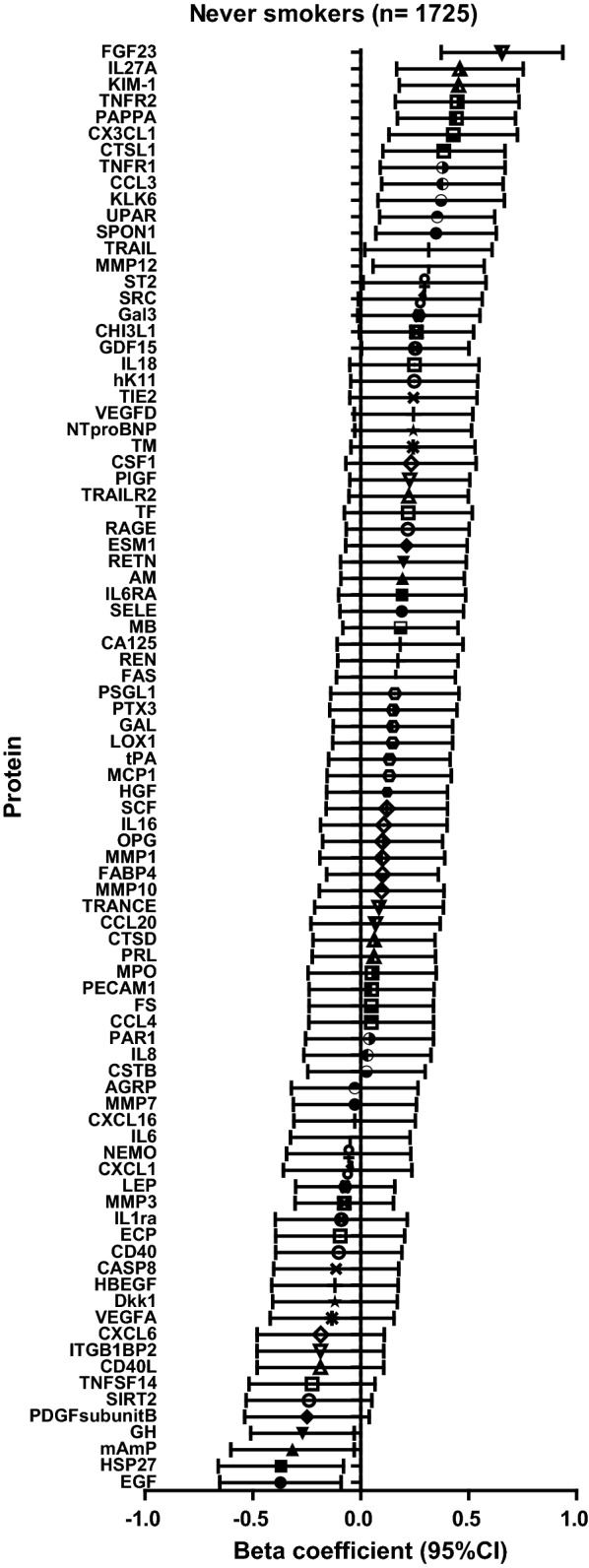
Association between blood cadmium and circulating proteins in never smokers (n = 1725)

Twenty proteins were significantly associated with B-Cd in never smokers (p < 0.05) (Table 2). B-Cd was inversely associated with four proteins and positively associated with 16 proteins. Fibroblast growth factor 23 (FGF-23) had the strongest association with B-Cd (beta coefficient: 0.66, 95% CI: 0.37–0.94, p = 5.0 × 10^−6^), Fig. 2. Of the 20 proteins with p < 0.05, 15 proteins passed the threshold for multiple testing and were also tested in long-term former smoker.

**Table 2:** Association between cadmium in blood and circulating proteins in never smokers and results from replication in long term former smokers

Protein	Never smokers (n = 1725)				Long-term former smokers (n = 782)			
	Beta	95% L	95% H	P	Beta	95% L	95% H	P
EGF	− 0.37	− 0.65	− 0.09	0.010	− 0.04	− 0.50	0.41	0.852
HSP27	− 0.37	− 0.66	− 0.08	0.013	0.09	− 0.37	0.56	0.690
mAmP	− 0.32	− 0.60	− 0.03	0.031				
GH	− 0.27	− 0.51	− 0.03	0.028				
GDF15	0.25	0.01	0.50	0.047				
ST2	0.30	0.01	0.58	0.041				
TRAIL	0.32	0.02	0.61	0.036				
MMP12	0.32	0.06	0.57	0.017	0.56	0.17	0.95	0.005
SPON1	0.35	0.07	0.63	0.014	0.19	− 0.24	0.62	0.380
UPAR	0.36	0.09	0.62	0.009	0.44	0.04	0.83	0.031
KLK6	0.37	0.08	0.67	0.013	0.36	− 0.08	0.79	0.108
CCL3	0.38	0.10	0.66	0.009	0.43	0.01	0.85	0.044
TNFR1	0.38	0.09	0.67	0.010	0.37	− 0.06	0.80	0.087
CTSL1	0.39	0.10	0.67	0.008	0.57	0.15	0.99	0.008
CX3CL1	0.43	0.13	0.73	0.005	0.45	0.01	0.88	0.044
PAPPA	0.44	0.17	0.72	0.001	0.12	− 0.30	0.53	0.584
TNFR2	0.45	0.16	0.73	0.002	0.52	0.09	0.94	0.017
KIM-1	0.45	0.18	0.73	0.001	0.78	0.36	1.20	3.2 × 10^−4^
IL27A	0.46	0.17	0.75	0.002	0.38	− 0.05	0.80	0.082
FGF23	0.66	0.37	0.94	5.0 × 10^−6^	0.71	0.29	1.13	0.001

95% L and 95% H indicate confidence limits for Beta

### Cadmium and protein biomarkers in long-term former smokers

**Fig. 3:**
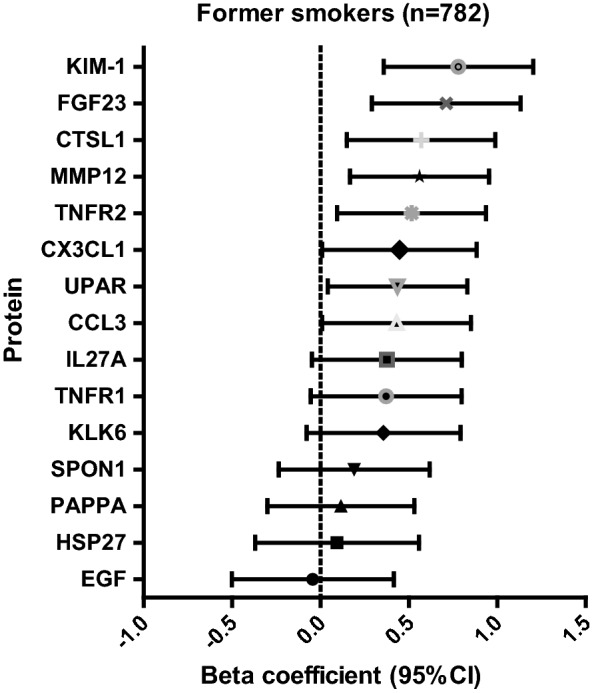
Associations between blood cadmium and circulating proteins in former smokers (n = 782)

Of the 15 proteins, eight (kidney injury molecule-1 (KIM-1), FGF-23, tumor necrosis factor receptor-2 (TNFR2), matrix metalloproteinase-12 (MMP-12), cathepsin L1, urokinase plasminogen activator receptor (UPAR), C-C motif chemokine-3 (CCL3), and chemokine C-X3-C motif (CX3CL1) were significantly associated with B-Cd (Table 2; Fig. 3). KIM-1 was found to have the strongest association with B-Cd (beta coefficient: 0.78, 95% CI: 0.36–1.20, p = 3.2 × 10^−4^), Fig. 3. We also adjusted the results for fiber intake, since fiber is an important source of dietary cadmium. The relationships between B-Cd and the eight proteins were still significant, except for CCL3, which became non-significant after adjustment for fiber intake.

### Cadmium, protein biomarkers and incidence of CVD in never smokers

Eight proteins (KIM-1, FGF-23, TNFR2, MMP-12, cathepsin L1, UPAR, CCL3, and CX3CL1) were significantly associated with B-Cd both in never and long-term former smokers. These proteins were explored for their relationship with incidence of CVD (Table 3).

**Table 3:** Hazards ratio (HR) for incident CVD (n = 224, 13.1%) in 1705 never smokers, expressed per 1 SD increment of protein biomarkers

Protein	Model 1^a^	P	Model 2^b^	P
KIM-1	1.29 (1.12–1.48)	< 0.001	1.16 (1.01–1.34)	0.033
FGF23	1.18 (1.03–1.36)	0.015	1.10 (0.96–1.27)	0.177
MMP12	1.29 (1.12–1.49)	< 0.001	1.23 (1.06–1.43)	0.006
CTSL1	1.28 (1.11–1.47)	0.001	1.22 (1.05–1.41)	0.007
TNFR2	1.25 (1.09–1.44)	0.001	1.16 (1.01–1.34)	0.036
UPAR	1.32 (1.14–1.54)	< 0.001	1.26 (1.08–1.47)	0.003
CCL3	1.15 (1.01–1.30)	0.037	1.06 (0.92–1.22)	0.441
CX3CL1	1.14 (1.01–1.30)	0.046	1.17 (1.02–1.33)	0.021

^a^Model 1: adjusted for age and sex. Hazard ratios (95% confidence interval) in all models

^b^Model 2: adjusted for age, sex, diabetes, systolic blood pressure, HDL, LDL, use of blood pressure, use of lipid-lowering drugs, and waist circumference

There were 224 incident events of CVD in 1705 never smokers (13.1%) during a mean follow up of 19.4 (SD: 4.6) years. All eight proteins were associated with an increased risk for CVD after adjustments for age and sex. The increased risk remained significant for KIM-1, UPAR, MMP12, cathepsin L1, TNFR2, and CX3CL1 after adjustments for cardiovascular risk factors (i.e., diabetes, systolic blood pressure, blood lipids, use of blood pressure or lipid-lowering medications and waist circumference).

## Discussion

Several studies from the recent years suggest that cadmium could cause atherosclerosis and CVD. However, the mechanistic link between cadmium and CVD is unclear. Cadmium has been associated with inflammation [11, 12, 22], inhibition of vascular cell proliferation [13], as well as apoptosis, necrosis [23] and increased expression of proteolytic enzymes, all of which could contribute to increased cardiovascular risk. Proteomics is a feasible method to identify biomarkers that are affected by the exposure of cadmium. The present study identified eight plasma proteins that were increased in never smokers and long-term former smokers with raised B-Cd levels. Six of them were also associated with increased incidence of CVD after adjustments for several risk factors. The results suggest that these proteins could be related to the pathogenic pathways linking cadmium to CVD.

Atherosclerosis is the underlying primary pathological process of CVD. Atherosclerotic plaques with a vulnerable phenotype are the plaques causing clinical events [24]. An accumulating body of data indicates that cadmium exposure is associated with the development and growth of atherosclerotic plaques [9] and the process leading to plaque rupture, myocardial infarction and ischemic stroke [7, 10, 24]. It has been shown that the cadmium content in symptomatic carotid atherosclerotic plaques is 50-fold higher than in blood [14]. Inflammation is a key mechanism in the atherosclerotic process and data indicate that cadmium is associated with the density of inflammatory macrophages in rupture-prone parts of human symptomatic carotid plaques [24]. CX3CL1, also known as fractalkine, initiates recruitment of monocytes in the atherosclerotic plaque and has been associated with plaque rupture, unstable angina pectoris and atherosclerosis at all stages [25, 26]. Hence, CX3CL1 could potentially be a mediator of the adverse effects of cadmium.

Cathepsin L1 is another biomarker associated with cadmium as well as incidence of cardiovascular events. Cathepsin L1 is a proteolytic enzyme which could degrade components of the extracellular matrix, such as elastin and type 1 and 2 collagen [27]. Cathepsin L1 has been shown associated with atherosclerotic plaques and plaque instability [28]. A study of vulnerable carotid plaque reported significantly higher cathepsin L concentrations in the upstream area where plaque rupture occurs most [29]. In a cohort of older adults, a significant association between cathepsin L1 and cardiovascular mortality was reported [30]. Our results indicate that cadmium exposure is associated with increased cathepsin L1 in plasma, which in turn could increase risk of plaque rupture and CVD.

UPAR was significantly associated with cadmium in this study. UPAR is a membrane-bound protein that is highly expressed in macrophages in symptomatic atherosclerotic plaques and is associated with fibrinolysis, cell migration, and matrix degeneration [31, 32]. It is also released from the cell surface into blood in a soluble form. In the present cohort the soluble form was found to be associated with B-Cd in never-smokers and with increased risk for carotid plaques and CVD [33, 34]. Soluble UPAR was measured by ELISA in those studies. Hence, the finding in the present study of a positive association between blood cadmium and UPAR has been corroborated by previous studies using a different technique. We are not aware of any animal experiments of cadmium exposure and its effect on plasma UPAR. However, a study of gastric cancer cells reported that cadmium induced increased expression of UPAR [35].

Cadmium has been demonstrated causing endothelial cell death and disruption of the functional integrity of the endothelium [14]. The permeability of the endothelium increases and blood vessels could therefore be more susceptible to lipid accumulation and inflammation [23]. MMP12, a member of matrix metalloproteinases family, has significant elastolytic activity, and has been linked to large artery stroke [36] as well as aortic abdominal aneurysms [37], and these outcomes have also been associated with raised B-Cd [7, 10, 38]. The proteolytic activity of MMP12 could further decrease the integrity of the endothelium and increase the risk of atherosclerosis and plaque rupture. To our best knowledge, there are no published data of associations between B-Cd and MMP12 in plasma.

A major proportion of cadmium in humans accumulates in the kidneys and it is well known that cadmium has adverse tubulointerstitial and glomerular effects. Several of the significant findings in this study were for biomarkers that previously have been related to renal diseases. For example, KIM-1 is considered as a sensitive and specific marker for renal proximal tubule damage [39]. A study of a population exposed to high cadmium concentrations reported significant relationships between urinary KIM-1 and cadmium in blood or urine [40] and urinary cadmium was correlated with urinary KIM-1 in a study of 109 kidney donors [41]. The relationship between cadmium and urinary KIM-1 is also reported from experimental studies of rats [42, 43]. To our knowledge, there are no previous studies of blood cadmium and plasma KIM-1 in humans. KIM-1 was also associated with incidence of CVD in this study. Hypothetically, this could be explained by increased risk of developing chronic kidney disease. Alternatively, the increased risk of CVD could be related to the effects of KIM-1 on immune cell activation [44].

FGF23 is a regulator of phosphate homeostasis, which inhibits renal tubular phosphate transport [45, 46]. It has been proposed that FGF23 may be responsible for the phosphaturic actions of cadmium [47]. Cross-sectional and population-based prospective studies have showed that FGF23 is a risk factor for low renal function and incident chronic kidney disease [48, 49]. B-Cd was significantly associated with FGF23 both in the never smokers and the long-term former smokers. Our results are supported by experimental studies of mice, which showed that administration of cadmium is followed by increased plasma FGF23 [47]. Altogether, the results from this study indicate that cadmium could have adverse effects on kidney function even in non-smokers with very low cadmium concentrations.

### Strengths and limitations

Our study used a proteomics approach with 88 circulating proteins and B-Cd levels from a well-established community-based cohort. Proteins with significant relationships in both the never smokers and the long-term former smokers were tested with respect to incidence of CVD. Smokers often have several-fold increased cadmium levels, compared to non-smokers. Smoking is also a major cause of raised inflammatory proteins and a major risk factor for CVD. The fact that the study sample was a cohort of never smokers, with replication in long-term former smokers, is a major strength of this study. The long-term former smokers had been smoke free for more than 15 years, and the pro-inflammatory effects of smoking should be substantially reduced after this time. The B-Cd levels were similar in the never smokers and the long-term former smokers.

The concentrations of B-Cd are comparable to other results from other Swedish cohorts [24, 50], but the mean values and range of B-Cd will obviously be lower when smokers are excluded. However, B-Cd was significantly associated with several plasma proteins, even though the concentrations were low. Hence, cadmium seems to have adverse health effects even at low concentrations [50].

Even though a wide range of plasma proteins were analyzed in this study, there are still many plasma proteins that potentially could mediate the effects of cadmium. Hence, more studies are needed to explore the effects of cadmium on the proteome. The proteins were presented as arbitrary units, based on real-time PCR quantification cycles. Hence, the relative concentrations were determined, but not the absolute values. This is a limitation of the study. However, the study cohort is from the general population and the distributions of proteins could therefore, by definition, be regarded as normal.

## Conclusions

We identified eight potential biomarkers of CVD and kidney injury associated with B-Cd. The results suggest pathways for the previously shown associations between cadmium exposure and incidence of cardiovascular disease and kidney injury.

## Abbreviations

AU: arbitrary units; B-Cd: blood cadmium;BMI: body mass index; CVD: cardiovascular disease; FDR: false discovery rate; HDL: high density lipoprotein; HR: hazard ratios; hsCRP: high-sensitive C-reactive protein; ICD: International Classification of Diseases; LDL: low-density lipoprotein; LOD: limit of detection; MDC-CC: the Malmö Diet and Cancer-cardiovascular cohort; NHANES: the National Health and Nutrition Examination Study; NPX: normalized protein expression; SD: standard deviations; SHDR: the Swedish Hospital Discharge Register.

### Proteins

AGRP: agouti-related protein; AM: adrenomedullin; BetaNGF: beta-nerve growth factor; BNP: natriuretic peptides B; CA-125: ovarian cancer-related tumor marker CA 125; CASP-8: caspase-8; CCL20: C-C motif chemokine 20; CCL3: C-C motif chemokine 3; CCL4: C-C motif chemokine 4; CD40: CD40L receptor; CD40L: CD40 ligand; CHI3L1: chitinase-3-like protein 1; CSF-1: macrophage colony-stimulating factor 1; CSTB: cystatin-B; CTSD: cathepsin D; CTSL1: cathepsin L1; CX3CL1: fractalkine; CXCL1: C-X-C motif chemokine 1; CXCL16: C-X-C motif chemokine 16; CXCL6: C-X-C motif chemokine 6; DKK-1: dickkopf-related protein 1; ECP: eosinophil cationic protein; EGF: epidermal growth factor; EN-RAGE: protein S100-A12; ESM-1: endothelial cell-specific molecule 1; FABP4: fatty acid-binding protein, adipocyte; FAS: tumor necrosis factor receptor superfamily member 6; FGF-23: fibroblast growth factor 23; FS: follistatin; GAL: galanin peptides; Gal-3: galectin-3; GDF-15: growth/differentiation factor 15; GH: growth hormone; HB-EGF: heparin-binding EGF-like growth factor; HGF: hepatocyte growth factor; hK11: kallikrein-11; HSP27: heat shock 27 kDa protein; IL-16: interleukin-16; IL-18: interleukin-18; IL-1RA: interleukin-1 receptor antagonist protein; IL-4: interleukin-4; IL-6: interleukin-6; IL-6RA: interleukin-6 receptor subunit alpha; IL-8: interleukin-8; IL27-A: interleukin-27 subunit alpha; ITGB1BP2: melusin; KIM-1: kidney injury molecule-1; KLK6: kallikrein-6; LEP: leptin; LOX-1: lectin-like oxidized LDL receptor 1; mAmp: membrane-bound aminopeptidase P; MB: myoglobin; MCP-1: monocyte chemotactic protein 1; MMP-1: matrix metalloproteinase-1; MMP-10: Matrix metalloproteinase-10; MMP-12: matrix metalloproteinase-12; MMP-3: matrix metalloproteinase-3; MMP-7: matrix metalloproteinase-7; MPO: myeloperoxidase; NEMO: NF-kappa-B essential modulator; NTproBNP: N-terminal pro-B-type natriuretic peptide; OPG: osteoprotegerin; PAPPA: pappalysin-1; PAR-1: proteinase-activated receptor 1; PDGF subunit B: platelet-derived growth factor subunit B; PECAM-1: platelet endothelial cell adhesion molecule; PlGF: placenta growth factor; PRL: prolactin; PSGL-1: P-selectin glycoprotein ligand 1; PTX3: pentraxin-related protein PTX3; RAGE: receptor for advanced glycosylation end products; REN: renin; RETN: resistin; SCF: stem cell factor; SELE: E-selectin; SIRT2: SIR2-like protein 2; SPON1: spondin-1; SRC: proto-oncogene tyrosine-protein kinase Src; ST2: ST2 protein; t-PA: tissue-type plasminogen activator; TF: tissue factor; TIE2: angiopoietin-1 receptor; TM: thrombomodulin; TNF-R1: tumor necrosis factor receptor 1; TNF-R2: tumor necrosis factor receptor 2; TNFSF14: tumor necrosis factor ligand superfamily member 14; TRAIL: TNF-related apoptosis-inducing ligand; TRAIL-R2: TNF-related apoptosis-inducing ligand receptor 2; TRANCE: TNF-related activation-induced cytokine; U-PAR: urokinase plasminogen activator surface receptor; VEGF-A: vascular endothelial growth factor A; VEGF-D: vascular endothelial growth factor D.
